# Event-Based Surveillance in mass gatherings: a global scoping review on effectiveness, scope, and lessons learned

**DOI:** 10.3389/fpubh.2026.1762219

**Published:** 2026-02-17

**Authors:** Farida Abougazia, Basma M. Saleh, Sungsoo Chun

**Affiliations:** School of Science and Engineering, Institute of Human Health and Human Ecology, The American University in Cairo, Cairo, Egypt

**Keywords:** epidemic intelligence, Event-Based Surveillance, infectious disease monitoring, mass gatherings, public health surveillance

## Abstract

**Introduction:**

Mass gatherings (MGs) attract large populations in specific places and times ranging from religious pilgrimages and cultural festivals to international sporting events. These events can drive rapid disease transmission, cross-border spread, and strain health systems requiring robust surveillance systems to detect health threats in real time. Event-Based Surveillance (EBS) offers a proactive approach to detecting such threats through systematic detection of signals. The aim of this scoping review is to assess global experiences of EBS during MGs focusing on geographical distribution, effectiveness, strategies, scope, and identifying lessons learned.

**Methods:**

A scoping review was conducted using PRISMA-ScR 2018 checklist and the Population, Concept, Context framework. All study types without time limits were included. Searches in PubMed, Scopus, ProQuest, and gray literature from WHO and CDC were screened. Two independent reviewers screened titles, abstracts and full texts then extracted data on study characteristics, surveillance design, scope, and outcomes.

**Results:**

From 1,469 publications, 28 were included where sportive events represented 39%, religious events represented 21%, cultural and political events 7% and others (33%) covered mixed or all MGs types. Publications included 50% peer-reviewed studies, 43% gray literature and 7% theses. EBS was reported effective in 75% of studies, partially effective in 11% and unspecified in 4 (14%) with one study highlighting evaluation gaps. Most publications (64%) integrated EBS with indicator-based and/or other surveillance while 36% applied standalone EBS. Scope was infectious-disease focused in 50% of studies with fewer all-hazard applications (39%) and 11% addressed specific areas such as bioterrorism. Multisectoral collaboration was the main best practice (46%) and coordination gaps represented the leading challenge (29%).

**Discussion:**

The included literature frequently describes EBS in MGs as effective, but the evidence base is limited and standardized evaluation criteria is needed. EBS in MGs is generally effective but under-represented in research. Future priorities include strong multisectoral collaboration, integrated surveillance systems, EBS evaluation platforms development and all-hazard coverage to ensure timely detection and preparedness for international mass gatherings.

## 1 Introduction

Mass gathering events are events that attract high numbers of participants to one particular location for a specific duration of time to attend or participate in an event. They can be categorized into spontaneous or planned events where spontaneous mass gathering events occur without prior preparations and can be further divided into unknown or unplanned events, such as refugee camps and protests. On the other hand, planned mass gathering events involve prior preparations and can be further divided into one-off events such as celebrations, royal weddings, and World Cup victory celebrations or recurrent ones that can either occur at changing locations, such as the Olympics and the Football World Cup or occur at the same location, like Hajj in Saudi Arabia and Arbaeen visit in Iraq. As shown in the previous categorization, mass gathering events can largely differ in nature but mainly lie under religious, sportive, political or cultural events (1).

Taking the religious mass gatherings as an example of annual mega mass gathering events such as Hajj (pilgrim) in Kingdom of Saudi Arabia, Arbaeen pilgrim in Iraq and Maha Kumbh Mela in India. These three events alone attract annually up to two, 21 and 120 million people from all over the globe, respectively (2).

Such large-scale mass gatherings raise significant public health risks including the spread of communicable diseases and exacerbating existing non-communicable diseases which consequently, burdens healthcare systems of not only the hosting countries, but also the home countries of the participants as well. This leads to a crucial need for these countries to have a strong and proactive public health surveillance strategy which is essential to enable early detection and rapid response to emerging health hazards (1).

Public health surveillance is defined as the continuous, systematic collection, analysis, and interpretation of health-related data needed for the planning, implementation, and evaluation of public health practices. This process serves as an early warning system for anticipated outbreaks, enables monitoring and evaluation of the impact of health interventions, helps track progress toward specified health goals and clarifies the epidemiology of health issues to inform public health decision-making (3).

Event Based Surveillance (EBS) is a form of surveillance *embedded within epidemic intelligence and public health intelligence systems*, and defined as the organized approach to the detection, *triage, verification* and reporting of ‘signals,’ which is information that may represent events of public health importance, often through channels outside of routine surveillance systems which is crucial for early warning systems. These channels can be media, social media, community or any other source of health-related information (4). EBS is considered a powerful approach toward the enhancement of early and real time outbreaks detection and response (5).

This scoping review aims to map and summarize mass gatherings EBS experience across global context. In addition, it documents global geographical distribution of EBS research and Implementation experience. This aims to identify best practices and challenges in conducting EBS for early detection of outbreaks or any other health-related hazard and to provide experts overview regarding recommendations and next steps for strengthening global mass gathering EBS context. As a scoping review, the aim is to map the available evidence, clarify key concepts, and identify research gaps, rather than to appraise the quality of evidence or synthesize quantitative measures of effectiveness.

## 2 Methodology

### 2.1 Approach

This scoping review adopted the PCC (Population, Concept, Context) Framework and is reported through the PRISMA extension for scoping reviews (PRISMA-ScR) guidelines published in 2018. PRISMA-P was initially used during protocol development, but the completed review aligns with PRISMA-ScR reporting standards (6).

- Population: Global mass gathering (MG) events that require specialized surveillance.
- Concept: Event-Based Surveillance (EBS) in mass gatherings.
- Context: Global.

### 2.2 Eligibility criteria

#### 2.2.1 Study inclusion criteria

Studies were included if they:

- Described or evaluated EBS systems implemented during MG events
- Included any study design whether quantitative and qualitative
- Included any publication types
- Were published in English and available in full text
- Had any date of publication (no time restrictions)

#### 2.2.2 Study exclusion criteria

Studies were excluded if they:

- Focused solely on other surveillance types (e.g., Indicator-Based Surveillance, syndromic surveillance)
- Described laboratory testing or preparedness plans without reference to EBS
- Were review articles, books, editorials, or non-English language publications without available translations

### 2.3 Information sources

Three electronic databases (Scopus, ProQuest, PubMed) were searched. Gray literature was retrieved from the World Health Organization’s Institutional Repository for Information Sharing (WHO IRIS) and the Centers for Diseases Prevention and Control (CDC)^1^ where the same core concepts (Event-Based Surveillance, epidemic intelligence, mass gatherings) adapted to each platform’s search interface.

### 2.4 Search strategy

A comprehensive Boolean search strategy combining relevant keywords and synonyms using “AND” and “OR” was employed. No time filters were applied during the initial search phase to maximize retrieval sensitivity though English language restriction was done.

**Table:** 

Database	Search query
PubMed	(“Event-Based Surveillance” OR “EBS” OR “Epidemic Intelligence” OR “Infectious Disease Monitoring”) AND (“Mass Gathering” OR “Mass Gatherings” OR “Mass Gathering Events” OR “Big Event” OR “Big Events” OR “Hajj” OR “Arbaeen” OR “Olympics” OR “FIFA World Cup” OR “Kumbh Mela”)
ProQuest	(“Event-Based Surveillance” OR “EBS” OR “Epidemic Intelligence” OR “Infectious Disease Monitoring”) AND (“Mass Gathering” OR “Mass Gatherings” OR “Mass Gathering Events” OR “Big Event” OR “Big Events” OR “Hajj” OR “Arbaeen” OR “Olympics” OR “FIFA World Cup” OR “Kumbh Mela”)
Scopus	(“Event-Based Surveillance” OR “EBS” OR “Epidemic Intelligence” OR “Infectious Disease Monitoring”) AND (“Mass Gathering” OR “Mass Gatherings” OR “Mass Gathering Events” OR “Big Event” OR “Big Events” OR “Hajj” OR “Arbaeen” OR “Olympics” OR “FIFA World Cup” OR “Kumbh Mela”)
WHO & CDC	“Event-Based Surveillance” AND “Mass Gathering”

The search was conducted for all fields using the basic search with no filters applied for all databases. Details of the search records, such as the date of the search, database, keywords, number of studies identified, and the number of eligible studies were appropriately documented.

### 2.5 Study records

#### 2.5.1 Data management

References were managed using Rayyan AI software to store citation records, remove duplicates and blind screening process. Reference lists were also managed using Zotero.

#### 2.5.2 Selection process

Two independent reviewers screened all titles and abstracts against the eligibility criteria then full-text screening followed for potentially relevant studies. Discrepancies among the reviewers were resolved by discussions till consensus was reached, if no consensus was reached, the third reviewer was consulted.

#### 2.5.3 Data extraction process

To account for heterogeneity in document type, records were classified as: (1) peer-reviewed implementation or evaluation studies, (2) peer-reviewed descriptive or analytical articles, (3) gray literature implementation or evaluation reports, and (4) gray literature guidance or technical documents. Data extraction and synthesis were stratified by document type where relevant (e.g., in reporting EBS effectiveness).

A standardized form was used to chart the following data:

- Bibliographic Information: Title, author(s), journal, type of publication, country, WHO region and publication year
- Study Characteristics: Design, MG event type, participants number
- EBS Dimensions: EBS effectiveness, duration, strategy, tools/sources and scope
- Key Outcomes: Best practices, gaps, recommendations

### 2.6 Data synthesis/charting

The extracted data were synthesized narratively and thematically covering the core objectives of the review. Findings were organized around the research questions with sections addressing EBS best practices, challenges, geographical patterns, and strategic recommendations.

### 2.7 Data analysis

We conducted a thematic analysis of the included publications identifying patterns using Microsoft Excel. We extracted the main recurring themes under the main domains in our review. Themes emerged from the data through comprehensive reviews allowing for merging and splitting findings when appropriate ensuring a structured synthesis of evidence.

## 3 Results

### 3.1 Overview and study characteristics

**Figure 1:**
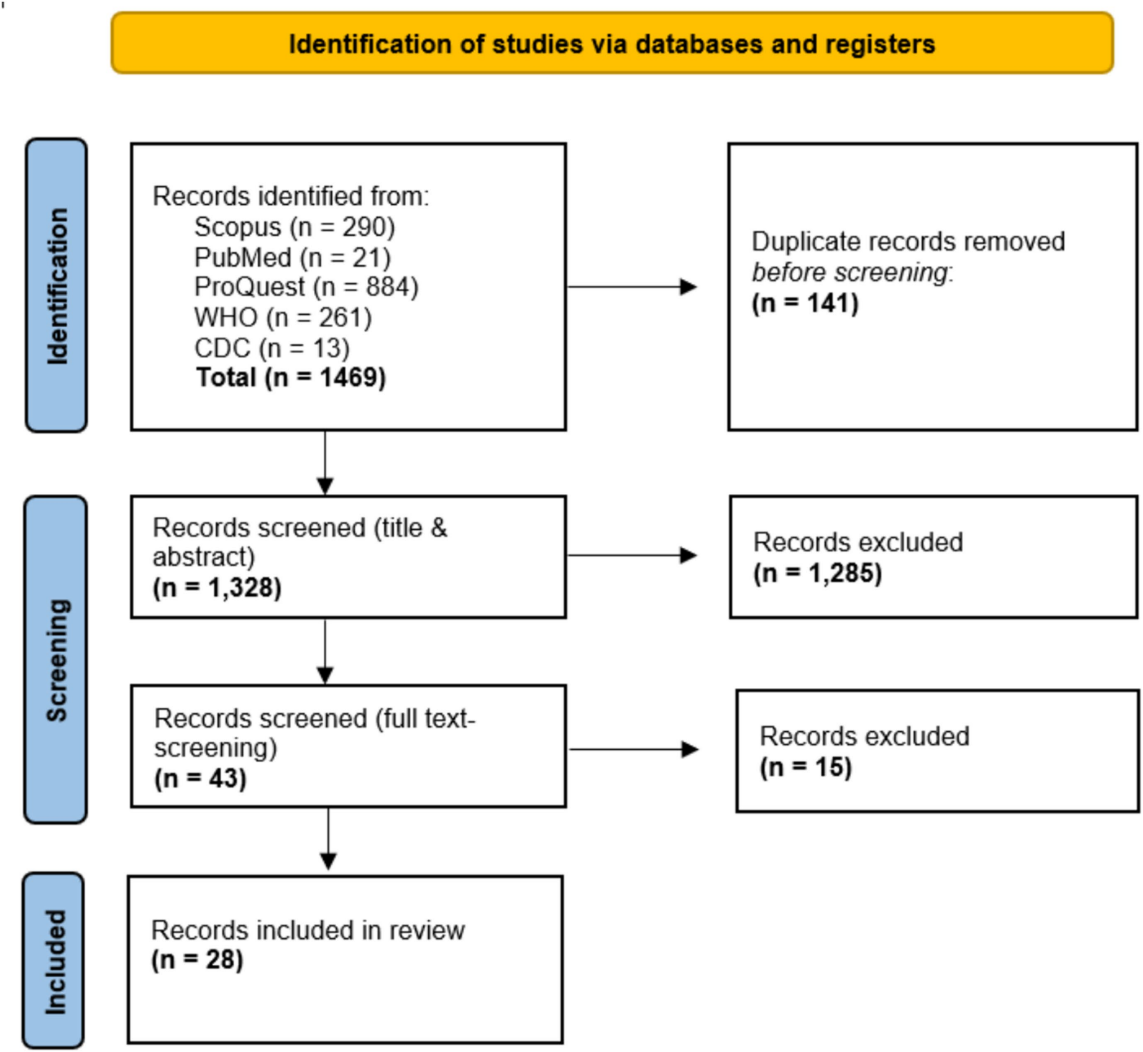
PRISMA flowchart of selection process for the scoping review of EBS during MG events.

As of March 30, 2025, we identified 1,469 studies, 141 were excluded as duplicates. Titles and abstracts screening for the remaining 1,328 studies resulted in the exclusion of 1,285 studies. The remaining 43 studies were subjected to full-text screening against the eligibility criteria leaving 28 included studies to be included (Figure 1). The study was completed on 27 May 2025.

A detailed data charting table (Supplementary Table) summarizing key extracted variables for all included documents including bibliographic details, MG event type, surveillance duration, signal sources, and verification workflows is provided in Supplementary material.

Among the 28 included records: 14 (50%) were peer-reviewed articles, 12 (43%) were gray literature (WHO reports, bulletins, technical guidance), and others were dissertation/thesis. When classified by document purpose: 11 (39%) were implementation or case-based reports, 5 (18%) were formal evaluations, and 12 (43%) were descriptive or guidance documents.

### 3.2 Geographical mapping of mass gathering events

Among the 28 included studies, Iraq (7–10), Saudi Arabia (11–14), and the United Kingdom (12, 15–17) were the most frequently represented, each appearing in four studies. Japan appeared in two studies (18, 19), while India (5), Qatar (20), Italy (21), Brazil (22), Lebanon (23), Spain (24), and South Africa (25) were each represented once. Several studies addressed multi-country or regional contexts across Europe, the Eastern Mediterranean, Africa, the Caribbean, and the Pacific (26–28). Two studies provided global guidance on EBS during mass gatherings (1, 29).

**Figure 2:**
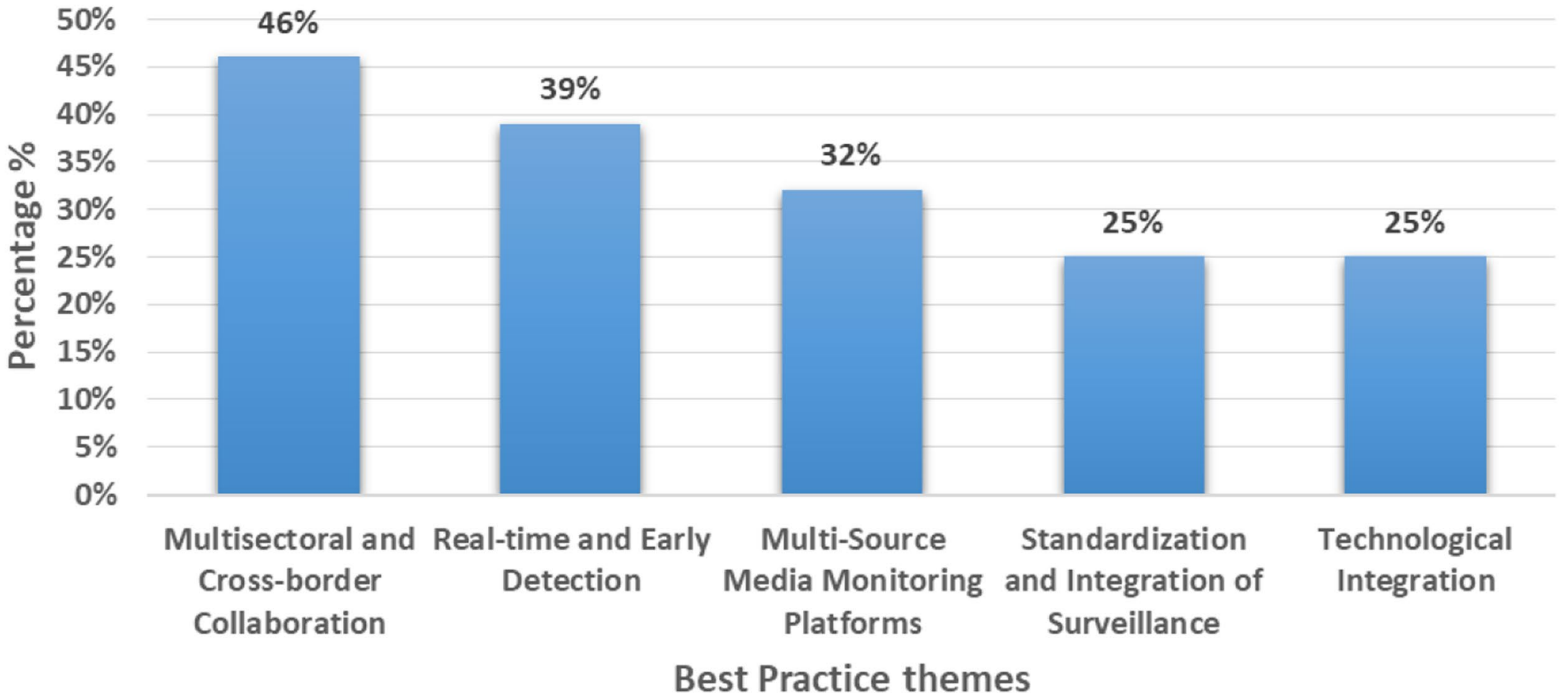
Key identified best practice themes.

**Figure 3:**
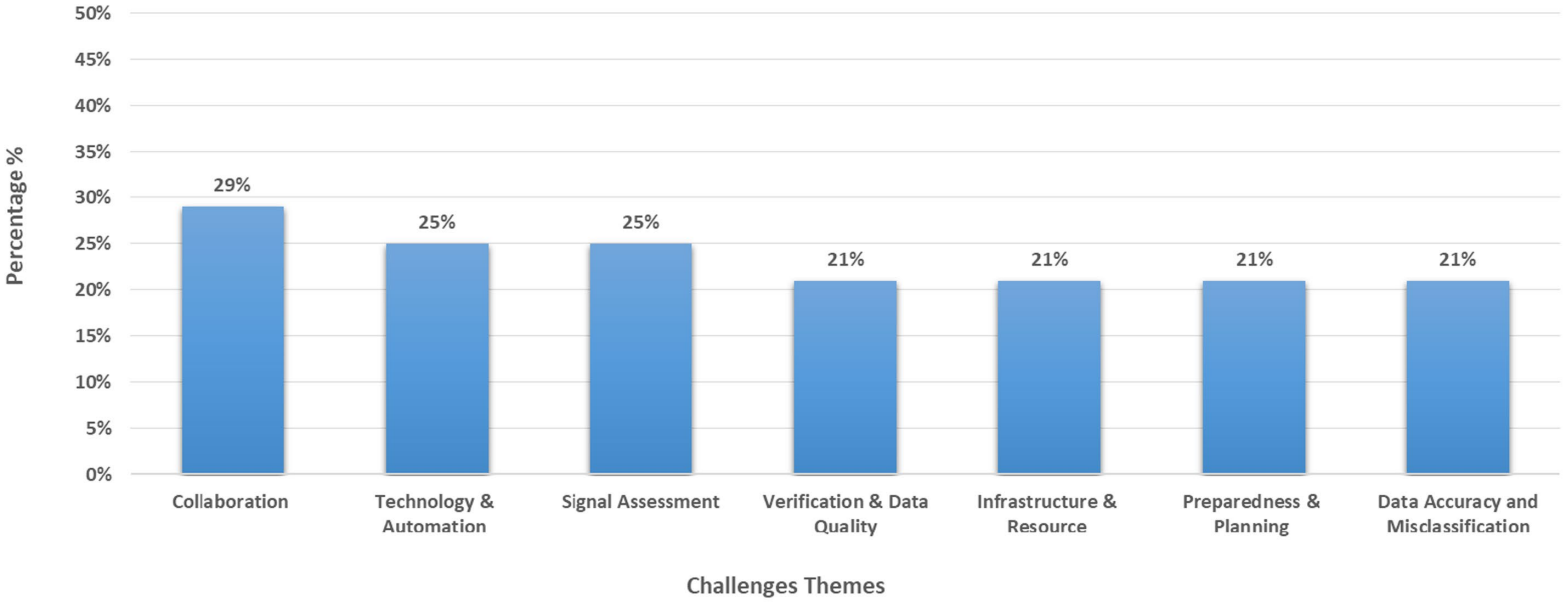
Key identified challenges themes.

**Figure 4:**
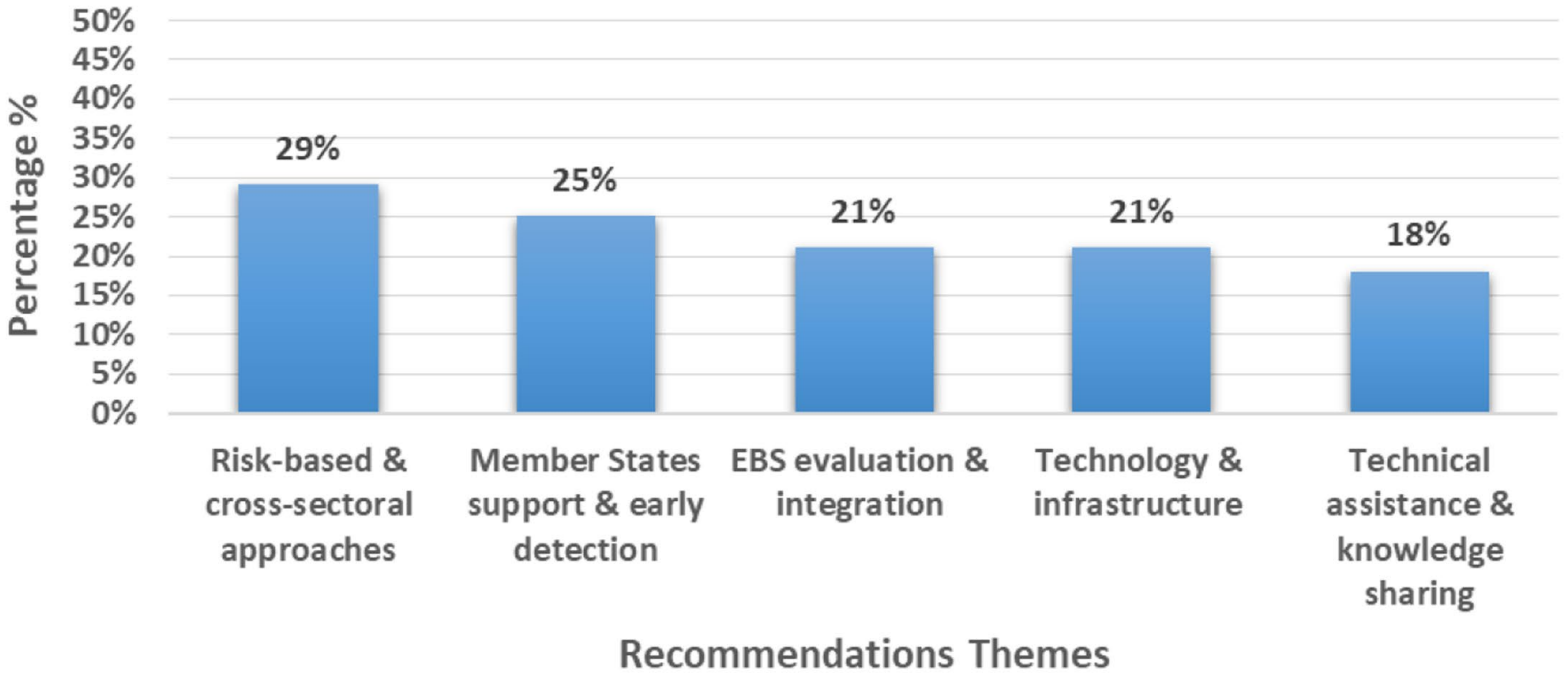
Key identified recommendations themes.

From the above figures, we can see that 11 studies (39%) came from just three countries (Saudi Arabia, Iraq, and the UK) while regional/multi-country contexts were covered in five studies (18%). Two studies (7%) provided global guidance (Figures 2–4 and Table 1).

**Table 1:** Included studies in this scoping review (*n* = 28).

No.	Study	MG event name	MG event type	Type of publication and study	Country
1	Aggrawala V., et al. 2020 (5)	Kumbh Mela	Religious event	Peer-Reviewed case-base study	India
2	Daniel K., et al. 2025 (30)	Cricket World Cup	Sportive event	Peer-Reviewed case-base study	Mix of six Caribbean countries
3	Yanagawa M., et al. 2022 (18)	Olympics and Paraolympic Games	Sportive event	Peer-Reviewed case-base study	Japan
4	Sallam M., et al. 2024 (20)	FIFA World Cup	Sportive event	Peer-Reviewed case-base study	Qatar
5	Severi E., et al. 2014 (15)	Olympics and Paraolympic Games	Sportive event	Peer-Reviewed evaluation study	United Kingdom
6	Kasamatsu, Ayu; et al. 2021 (19)	Olympics and Paraolympic Games	Sportive event	Peer-Reviewed case-base study	Japan
7	Riccardo F, et al. 2016 (21)	EXPO	Cultural event	Peer-Reviewed case-base study	Italy
8	WHO 2024 (13)	Multiple	Mix	Gray Literature (Quarterly bulletin)	EMR countries
9	WHO 2024 (11)	Hajj	Religious event	Gray Literature (Biweekly Bulletin)	Kingdom of Saudi Arabia
10	WHO 2024 (7)	Arbaeen	Religious event	Gray Literature (Biweekly Bulletin)	Iraq
11	Mantero S., et al. 2014 (25)	FIFA World Cup	Sportive event	Peer-Reviewed Article (Report)	South Africa
12	Johns J., et al. 2013 (16)	Olympics and Paraolympic Games	Sportive event	Peer-Reviewed evaluation study	United Kingdom
13	Economopoulou A., et al. 2014 (17)	Olympics and Paraolympic Games	Sportive event	Peer-Reviewed evaluation study	United Kingdom
14	Wolfe, Caitlin M. 2024 (8)	Internally Displaced People camps	Internally Displaced People Camps	Dissertation	Iraq
15	William SG, et al. 2021 (27)	Multiple	Mix	Accepted manuscript (Retrospective Analysis)	AFRO
16	Burkom H., et al. 2021 (32)	Multiple	Mix	Peer-Reviewed Article	Mix (United States of America and others)
17	Leal O, et al. 2017 (22)	FIFA World Cup	Sportive event	Peer-Reviewed case-base study	Brazil
18	Yom-Tov E, et al. 2014 (12)	Multiple	Mix	Peer-Reviewed Article	Mix (United Kingdom & Kingdom of Saudi Arabia)
19	Enderlein U, et al. 2018 (28)	Multiple	Mix	Gray Literature (WHO case report)	EUR countries
20	WHO 2015 (1)	All	All	Gray Literature (Technical Guidance)	All
21	WHO 2021 (29)	All	All	Gray Literature (Technical Guidance)	All
22	Schmidt T., et al. 2023 (31)	Union of European Football Associations (UEFA)	Sportive event	Gray Literature (WHO case report)	Mix (13 European countries)
23	Haddad N., et al. 2017 (23)	Sixth Francophone Games	Sportive event	Peer-Reviewed case-base study	Lebanon
24	WHO 2023 (24)	Malta Pride; Maspalomas Gay Pride	Cultural event	Gray Literature (WHO webinar)	Spain
25	WHO 2019 (9)	Arbaeen	Religious event	Gray Literature (Summary Report)	Iraq
26	WHO 2019 (10)	All	All	Gray Literature (Evaluation Report)	Iraq
27	WHO 2017 (13)	Hajj	Religious event	Gray Literature (Evaluation Report)	Kingdom of Saudi Arabia
28	WHO 2013 (14)	Hajj	Religious event	Gray Literature (Report)	Kingdom of Saudi Arabia

### 3.3 Mass gathering event types and scopes

Mass gathering Event types are distinguished within the included studies. Most documented events were either sporting events (39%, *n* = 11) or religious ones (21%, *n* = 6). Among the sportive events included are Olympic and Paralympic games as featured in five studies from Japan, and UK. This represents international multi-sport events with tens of thousands of participants and extensive international travel. FIFA World Cup Events and Cricket World Cup are also featured in four studies, and one study, respectively. Other sportive events included UEFA Euro 2020 and Francophone Games.

Cultural and political mass gatherings appeared in two studies (7% of the included studies). Example is internally displaced people camps which are featured in one study and it represents a unique mass gathering contexts with distinct epidemiological profiles including concentrated vulnerable populations, limited healthcare access, and water/sanitation challenges. Mixed/all MGs types appeared in nine studies (33%).

### 3.4 Event-Based Surveillance effectiveness and integration patterns

The 28 included studies provided descriptive and evaluative data on EBS effectiveness. Among the 28 included studies: 75% of studies (*n* = 21) reported EBS systems as effective in detecting signals, generating early warnings, or meeting stated surveillance objectives. 11% of studies (*n* = 3) described intermediate effectiveness with some limitations identified. 14% of studies (*n* = 4) did not provide explicit effectiveness assessments or acknowledged gaps in evaluation frameworks.

Guidance documents and high-level technical reports do not usually evaluate specific EBS implementations in the same way as field reports. Thus, effectiveness was reported separately by document type. Among the 15 evaluative records (implementation reports or evaluation studies): 80% (*n* = 12) described EBS systems as effective in detecting signals or meeting surveillance objectives, 13% (*n* = 2) reported intermediate effectiveness with limitations, and 7% (*n* = 1) did not provide an explicit assessment.

For the integration pattern, most publications (64%, *n* = 18) integrated EBS with indicator-based and/or other surveillance types, while (36%, *n* = 10) applied standalone EBS approaches. Integrated surveillance is reported when EBS systems operate alongside indicator-based surveillance (IBS) systems, syndromic surveillance, and routine surveillance structures, with coordinated signal management and joint review processes. On the other hand, standalone EBS is also reported as an independent surveillance function focused on media monitoring, social media signals, and informal reporting channels without formal integration into national routine surveillance systems.

### 3.5 Surveillance scope and all-hazards approach

There are various scopes reported for EBS implementations, revealing gradual expansion from disease-specific to broader all-hazards surveillance. Half (50%) of studies (*n* = 14) specifically targeted infectious diseases including vector-borne diseases, respiratory infections, foodborne illnesses, and pandemic threats while 39% of studies (*n* = 11) explicitly described all-hazards surveillance encompassing infectious diseases, non-communicable diseases, injuries, environmental hazards, heat-related illnesses, accidents, and emergencies. On the other hand, 11% of studies (*n* = 3) focused on specific threat areas including bioterrorism (study 3, Tokyo Olympics) and special disease concerns (study 13), prioritized disease surveillance at EU level for Olympics.

### 3.6 Technological platforms and tools

The 28 included studies described diverse technological approaches to use while implementing EBS, ranging from manual systems to advanced artificial intelligence-enabled platforms. EIOS (Epidemic Intelligence from Open Sources) developed by WHO is featured in studies 3, 4, 6, 10. This platform performs automated media scanning from diverse pools of news agencies with the focus of for public health threats related news. EIOS was increasingly integrated into mass gathering EBS with human expert validation required for signal assessment, triage, verification, risk assessment and response. MedISys Platform is a European Commission-developed tool and is featured in studies 7, 11, 23 for automated media monitoring and multilingual signal detection from official and unofficial sources which is very similar to EIOS.

Hazard Detection and Risk Assessment System is featured in study 19 as a platform for monitoring news media, social media, and blogs for public health threats, used during European cultural events. A Caribbean-specific mass gathering surveillance platform is featured in study 2 as another platform integrating electronic data collection forms, tablet-based field entry, alert notification mechanisms, and real-time reporting features.

ESSENCE and OpenESSENCE: Featured in study 16. Syndromic surveillance systems with modular deployment, shareable visualizations, automated queries, and cohort tracking capabilities. While Twitter and Bing Query Logs which are featured in study 18 are internet-based syndromic surveillance using statistical modeling for mass gathering surveillance during festivals and Hajj.

Participatory Surveillance Applications are also featured in study 17 including mobile application (Healthy Cup app, later Guardians of Health) enabling direct public reporting of health symptoms during FIFA World Cup 2014, generating 47,879 participatory surveillance posts.

### 3.7 Key thematic best practices

1. Multisectoral and Cross-border Collaboration was the most frequently cited practice, reported in 13 studies (5, 8–12, 14–17, 21, 23, 27). This involved coordination among Ministries of Health, WHO, ECDC, Gulf CDC, and civil society organizations. Notably, 25 of the 28 studies reported engagement of multiple stakeholders, with WHO involved in 17 studies and ECDC in 8.
2. Real-time and Early Detection systems such as alerts and dashboards were highlighted in 11 studies, enabling timely situational awareness and outbreak response through early warning mechanisms (1, 8, 11, 12, 15, 17, 18, 25, 28–30).
3. Multi-Source Media Monitoring Platforms appeared in 9 studies, leveraging tools like EIOS and MedISys to capture signals from both official and unofficial digital sources (5, 9, 10, 12, 15, 20, 22, 23, 31).
4. Standardization and Integration of Surveillance Systems was noted in 7 studies, emphasizing the alignment of EBS with existing indicator-based and routine surveillance structures (10, 14, 19, 23, 26, 29, 30).
5. Technological Integration ranging from basic digital tools to advanced AI-driven platforms was noted in 24 studies overall, with 7 specifically detailing high-tech enhancements such as mobile data entry, modular platforms, and validation models (9, 10, 13, 17, 26, 29, 31).

### 3.8 Key thematic challenges

The review identified seven recurring challenges that hinder the implementation of Event-Based Surveillance (EBS) during mass gatherings:

1. Coordination and Collaboration Issues were the most cited challenge (eight studies), involving cross-border communication gaps and lack of interagency coordination (1, 11, 12, 15, 16, 21, 26, 28).
2. Technology and Automation Gaps appeared in seven studies, where limited digital tools led to inefficient, manual data processes (1, 9, 10, 14, 23, 24, 29).
3. Signal Assessment and Data Processing challenges were also present in seven studies, including difficulties with evolving threat tracking and zero-reporting (1, 9, 14, 23, 24, 29, 31).
4. Verification and Data Quality issues emerged in six studies, often due to delays in validating media-derived signals (5, 12, 20, 24, 26, 30).
5. Infrastructure and Resource Constraints were reported in six studies, noting manual reporting systems, limited resources, and inconsistent implementation (9, 10, 12, 23, 24, 29).
6. Preparedness and Planning Issues cited in six studies, included limited training and the absence of structured risk frameworks (15, 16, 21, 24, 26, 28).
7. Data Accuracy and Misclassification also appeared in six studies, particularly involving false positives and inconsistent data formats (9, 10, 13, 24, 29, 31).

### 3.9 Key thematic recommendations and next steps

The review identified five thematic recommendations to strengthen Event-Based Surveillance (EBS) in mass gathering settings:

1. Risk-based approaches and cross-sectoral collaboration were the most emphasized, appearing in eight studies, promoting proactive planning and coordinated stakeholder engagement (5, 7, 14–16, 21, 31, 32).
2. Support to Member States and early detection was recommended in seven studies, stressing investment in preparedness systems and real-time detection capacity (5, 7, 17, 19, 20, 29, 30).
3. Evaluation and integration of EBS into national surveillance structures appeared in seven studies, advocating for regular performance reviews and workforce training (7, 14–16, 19, 20, 27).
4. Technology and infrastructure improvement was noted in six studies, recommending enhanced digital systems, private sector collaboration, and mobile tools for timely reporting (8, 11, 14, 19, 30, 32).
5. Technical assistance and knowledge sharing also cited in six studies, highlighted the need for training, research dissemination, and regional or global collaboration mechanisms (5, 8, 11, 19, 22, 28).

## 4 Discussion

Our scoping review covered relevant global publications including gray literature with no time constrictions. However, it ended up with 28 publications indicating limited research of EBS during mass gathering events globally (1, 33). This could be attributed to multiple factors: (1) the focus on English-language publications as an inclusion criterion, which may exclude relevant non-English or paywalled studies; (2) documentation bias where EBS activities during mass gatherings may be implemented but not formally documented or published in indexed sources; and (3) the concentration of published literature in certain geographic regions and event types, with limited research from lower-income countries despite frequent mass gathering events in these settings.

The 28 included publications came from diverse sources and in different formats from peer reviewed articles to webinar summaries. The predominance of WHO-authored gray literature compared to national health authorities is likely attributed to its greater capacity in terms of time and human resources to document EBS experiences.

### 4.1 Geographical patterns

Geographical patterns of the included studies show broad gap as 11 (39%, *n* = 11) studies come from just three countries (Saudi Arabia, Iraq, and the UK) while there is a significant underrepresentation of documented EBS experiences from: (1) countries in sub-Saharan Africa despite the frequent occurrence of major religious and cultural events; (2) Southeast Asia beyond Japan; (3) the Americas beyond Brazil and Caribbean region; and (4) lower-income and lower-middle-income countries. This highlights a geographic gap in the literature and underscores the need for broader global documentation efforts of EBS experiences during mass gatherings (1, 34).

### 4.2 Mass gathering events types

Most documented events were either sporting events or religious ones which probably reflects the higher public, political and media visibility of such international gatherings compared to other types of events such as cultural festivals or refugee camps.

Sports events attract large numbers of participants and create sustained transmission opportunities over the event period. Religious events on the other hand, represent the second most frequently documented event type, featured in six studies encompassing some of the world’s largest gatherings by attendance. Religious events require unique surveillance efforts due to (1) extended duration; (2) geographically dispersed participants across multiple sites; (3) populations including older adults and immunocompromised individuals; (4) varied access to healthcare; and (5) cross-border pilgrimage patterns.

Cultural events’ underrepresentation in literature may reflect lower epidemiological priority perception compared to sportive and religious events, though they merit greater surveillance attention given growing event scale and international attendance.

### 4.3 Surveillance scope, all-hazards approach, and integrated approach

While all-hazards approaches are increasingly adopted, infectious disease main focus remains dominant, suggesting that EBS frameworks have not fully integrated all public health threats comprehensively across all settings.

On the other hand, integrated disease surveillance approach reflects a growing recognition that EBS functions most effectively as component of existing comprehensive surveillance systems rather than an isolated function.

### 4.4 Best practices and challenges

The most frequently mentioned best practice was multisectoral and stakeholders’ collaboration for EBS surveillance reflecting the multidirectional support needed for early detection, communication, validation and eventually response to detected signals. This is similar to existing literature focusing on the importance of multisectoral stakeholders in EBS (34, 35). Simultaneously, it was also found to be a main challenge in many studies showing the great importance of collaboration efforts in EBS activities during mass gatherings.

The second most important aspect was technology integration as proper technology integration was mentioned as a best practice while poor technology integration was highlighted as a main challenge in seven studies each. This aligns with other studies and reviews (36, 37) which is expected to increase due to recent Artificial Intelligence technologies and the limited human resources which makes high technology integration an important aspect for EBS specially that EBS includes media and social media which are heavily connected with such technology. Real time detection and communication was highlighted in accordance with existing literature (38) and high technology integration helps minimizing the lag time needed for detection, assessment and reporting any signal.

These findings underscore the complexity of implementing EBS during mass gatherings and indicates the urgent need for improvements in stakeholders’ coordination, better data automation and proper data management to enhance public health surveillance efforts.

### 4.5 Recommendations and next steps

The recommendations synthesized in this review primarily reflect actions proposed within the included records, focusing on how EBS in mass gatherings could be strengthened in practice.

According to this review findings, proper EBS integration with other types of surveillance is the best way forward to increase the effectiveness of EBS and to minimize the effort and resources needed for it as it will be a routine task in the national systems. This is agreed upon by other studies as a key recommendation (39, 40).

Risk based approaches and multisectoral collaboration are very crucial for EBS activities along with continuous evaluation of the system (34, 35, 41, 42).

Deeper analysis to the common areas reveals five key common themes consistently emerged across best practices, challenges and recommendations indicating common priorities for strengthening EBS during MGs according to the included publications. Surveillance systems integration and data quality improvement was the most frequently cited theme highlighted. Technology and infrastructure followed highlighting the potential of maximizing digital tools and Artificial Intelligence (AI) in EBS activities. Cross-sectoral collaboration was also highlighted which shows its significance particularly in cross-border and multisectoral contexts which is very common in mass gatherings. Early detection and real-time analysis and capacity building were each identified in the selected studies emphasizing the value of timely signal capture which is the main objective of EBS and the sustained support to national systems performing EBS activities.

These recommendations provide a roadmap for institutionalizing, upscaling and sustaining EBS activities in the context of mass gatherings with a strong emphasis on risk based and cross-sectoral approaches, evaluation and integration of EBS systems, technological advancement and knowledge sharing.

### 4.6 Future research

We recommend based on our findings that vigorous future research is essential for comparative studies and evaluation of current EBS systems during mass gatherings in a standardized way to document legacy and provide evidence-based guidance for global EBS during mass gatherings.

### 4.7 Study limitations

The main limitation in this scoping review is that it included only open-access, English-language sources which accordingly excluded relevant non-English or paywalled studies. The second limitation is that the search was limited to three databases and two gray literature sources, omitting other possible sources such as national Ministry of Health publications. Documentation bias might also add to these limitations due to lack of documentation of EBS even if it is already in place.

## 5 Conclusion

This scoping review presents the current state of literature on Event-Based Surveillance (EBS) during mass gathering events as of March 2025. While multisectoral collaboration and technological integration were commonly emphasized as strengths, gaps remain in coordination, data quality, and preparedness.

Across the included records, EBS in mass gatherings was frequently described as effective for detecting signals and generating early warnings. However, the evidence base remains limited by heterogeneous, author-reported assessments and lack of standardized evaluation frameworks. This scoping review highlights the need for rigorous, comparative evaluation of EBS systems using consistent outcome metrics.

Further research is needed to evaluate EBS systems using standardized form, strengthen risk-based approaches, and support sustainable implementation. As mass gatherings continue to evolve, ongoing review of emerging evidence is essential to guide public health planning and policies.

## Data Availability

The original contributions presented in the study are included in the article and supplementary material. Further inquiries can be directed to the corresponding author.

## Footnotes

1
https://stacks.cdc.gov/
